# Prevalence of Pelvic Inflammatory Disease in Sexually Experienced Women of Reproductive Age — United States, 2013–2014

**DOI:** 10.15585/mmwr.mm6603a3

**Published:** 2017-01-27

**Authors:** Kristen Kreisel, Elizabeth Torrone, Kyle Bernstein, Jaeyoung Hong, Rachel Gorwitz

**Affiliations:** 1 Division of STD Prevention, National Center for HIV/AIDS, Viral Hepatitis, STD, and TB Prevention, CDC.

Pelvic inflammatory disease (PID) is a clinical syndrome of the female reproductive tract characterized by inflammation of the endometrium, fallopian tubes, or peritoneum (*1*). PID occurs when microorganisms ascend from the vagina or cervix to the fallopian tubes and other upper genital tract structures (*1*). PID can result from untreated bacterial infections, including chlamydia and gonorrhea, and can lead to infertility, ectopic pregnancy, and chronic pelvic pain (*1*). Because there is no single diagnostic test for PID, clinicians rely on nonspecific signs and symptoms for diagnosis. The purpose of these analyses was to assess the burden of self-reported PID in a nationally representative sample using data from the National Health and Nutrition Examination Survey (NHANES) 2013–2014 cycle. Starting in 2013, NHANES female participants aged 18–44 years were asked about a lifetime history of PID diagnosis. Based on these data, the estimated prevalence of self-reported lifetime PID was 4.4% in sexually experienced women of reproductive age (18–44 years). The prevalence of self-reported lifetime PID was highest in women at increased risk, such as women reporting a previous sexually transmitted infection (STI) diagnosis. Stratified by race/ethnicity and having a previous STI diagnosis, non-Hispanic black (black) and non-Hispanic white (white) women reporting a previous STI diagnosis had nearly equal self-reported lifetime PID prevalence (10.0% versus 10.3%). However, the lifetime prevalence of PID among black women was 2.2 times that among white women if no previous STI was diagnosed (6.0% versus 2.7%). These findings suggest that PID is prevalent and associated with previous STI diagnoses; therefore, it is important for clinicians to screen female patients for chlamydia and gonorrhea to reduce the incidence of PID.

NHANES is a cross-sectional, complex, multistage survey designed to be nationally representative of the noninstitutionalized U.S. civilian population (https://www.cdc.gov/nchs/nhanes.htm). Participants undergo a medical examination and are interviewed in person, during which time questions regarding sexual and reproductive health are asked. In NHANES 2013–2014, a total of 1,444 women aged 18–44 years were interviewed and had a medical exam; the response rate was 71.1%. The 1,171 (81%) reproductive-aged female participants who responded “Yes” to the question, “Have you ever had vaginal, anal, or oral sex?” were defined as sexually experienced and were the focus of these analyses. Participants who responded “Yes” to the question, “Have you ever been treated for an infection in your fallopian tubes, uterus or ovaries, also called a pelvic infection, pelvic inflammatory disease, or PID?” met the case definition of a lifetime PID diagnosis. Having received a diagnosis of a previous STI was defined as having had a chlamydia or gonorrhea infection during the past 12 months or ever having had herpes, human papillomavirus, or genital warts. The prevalence of self-reported lifetime PID, prevalence ratios (PRs), and 95% confidence intervals (CIs) were estimated overall and by various characteristics. Associations were measured by use of the Rao-Scott chi-square test. All analyses were conducted using SAS statistical software (version 9.3) and accounted for the complex survey design and sampling weights. As such, these results are nationally representative. The mobile examination center exam sampling weights were used to weight the data. Population counts were estimated by multiplying weighted prevalence estimates by the average of the American Community Survey estimates during 2013–2014.

Among 1,171 sexually experienced reproductive-aged women in NHANES 2013–2014, the prevalence of self-reported lifetime PID was 4.4% (Table), indicating that approximately 2.5 million women aged 18–44 nationwide have received a diagnosis of PID in their lifetime (95% CI = 1.8–3.2 million). No significant differences existed in prevalence of a lifetime PID diagnosis by age, race/ethnicity, or socioeconomic factors, such as income-poverty ratio, current health insurance coverage, or having a current usual place for health care.

**TABLE:** Prevalence of self-reported lifetime pelvic inflammatory disease* among sexually experienced women^†^ aged 18–44 years (n = 1,171), by selected characteristics — National Health and Nutrition Examination Survey, United States, 2013–2014.

Characteristic	Sample size no.	Prevalence (%)^§^ (95% CI)	Prevalence ratio^¶^ (95% CI)
**Total**	**1,171**	**4.4 (3.1–5.7)**	**— (—)**
Age group (yrs) (p = 0.28)**			
18–24	327	2.9^††^ (0.8–5.0)	Ref (—)
25–29	185	4.6^††^ (1.4–7.9)	1.6 (0.6–4.1)
30–34	212	5.0^††^ (1.8–8.2)	1.7 (0.7–4.3)
35–39	209	3.5 (1.5–5.4)	1.2 (0.5–2.7)
40–44	238	6.7 (2.6–10.8)	2.3 (0.8–6.6)
Race/Ethnicity (p = NC)**			
White, non-Hispanic	436	4.4 (2.8–6.0)	Ref (—)
Black, non-Hispanic	245	6.8 (4.0–9.5)	1.5 (0.9–2.5)
Asian, non-Hispanic	130	0.0 (—)	— (—)
Mexican American	195	—^§§^ (—^§§^)	—^§§^ (—^§§^)
Education level (p = 0.21)**			
Less than high school	212	4.3^††^ (1.0–7.6)	Ref (—)
High school graduate/GED	243	3.1^††^ (1.1–5.2)	0.7 (0.3–1.8)
Some college/Associates degree	430	6.2 (3.2–9.2)	1.5 (0.6–3.7)
College graduate or above	286	3.0^¶¶^ (0.4–5.7)	0.7 (0.2–2.9)
Marital status (p = 0.56)**			
Married/Living with partner	618	5.0 (2.5–7.6)	Ref (—)
Divorced/Separated/Widowed	120	5.3^¶¶^ (0.6–9.9)	1.0 (0.4–3.0)
Never married	309	3.5 (1.3–5.8)	0.7 (0.3–1.9)
Income-poverty ratio*** (p = 0.50)**			
<150% FPL	500	5.1 (2.9–7.3)	Ref (—)
150%–299% FPL	244	4.7^††^ (1.0–8.4)	0.9 (0.3–2.5)
≥300% FPL	357	3.2^††^ (0.9–5.4)	0.6 (0.3–1.5)
Health insurance coverage (p = 0.20)**			
Covered	860	4.0 (2.5–5.5)	Ref (—)
Not covered	303	6.1 (2.6–9.6)	1.5 (0.7–3.2)
Has a usual place for health care (p = 0.87)**			
Yes	952	4.4 (2.6–6.1)	Ref (—)
No	219	4.7^††^ (1.2–8.3)	1.1 (0.4–2.9)
Type of place for usual health care (p = NC)**			
Doctor's office/HMO	655	3.8 (1.8–5.9)	Ref (—)
Clinic/health center	219	6.0^††^ (1.2–10.9)	1.6 (0.6–4.2)
Hospital outpatient department/ED	63	—^§§^ (—^§§^)	—^§§^ (—^§§^)
Age at sexual debut, in years (p = 0.0002)**			
≥18	475	2.7 (1.2–4.2)	Ref (—)
16–17	371	4.6 (1.7–7.5)	1.7 (0.7–4.3)
14–15	228	4.9 (2.5–7.4)	1.8 (0.9–3.6)
12–13	79	8.9 (3.3–14.5)	3.4 (1.4–8.5)
<12	18	23.6^¶¶^ (0.9–46.2)	8.6 (2.7–27.9)
Sexual orientation (p = NC)**			
Heterosexual	1,042	4.1 (2.8–5.4)	Ref (—)
Lesbian/Bisexual	102	8.7^††^ (2.3–15.1)	2.1 (0.9–4.8)
No. male lifetime vaginal sex partners (p = 0.0005)**			
1	292	2.5^¶¶^ (0.1–4.8)	Ref (—)
2–3	230	2.0^††^ (0.3–3.7)	0.8 (0.3–2.4)
4–9	400	4.1 (2.1–6.0)	1.7 (0.7–4.2)
≥10	249	8.7 (5.0–12.4)	3.6 (1.2–10.7)
Previous STI diagnosis^†††^ (p<0.0001)**			
No	978	3.1 (1.9–4.2)	Ref (—)
Yes	193	10.2 (6.0–14.3)	3.3 (1.9–5.7)

**Abbreviations:** CI = confidence interval; ED = emergency department; FPL = Federal Poverty Level; GED = General Educational Development certification; HMO = health maintenance organization; NC = not calculated; STI = sexually transmitted infection.

* Prevalence estimates based on response to the question “Have you ever been treated for an infection in your fallopian tubes, uterus or ovaries, also called a pelvic infection, pelvic inflammatory disease, or PID?”

^†^ Based on a response of “Yes” to the question “Have you ever had vaginal, anal, or oral sex?”

^§^ Estimates were weighted to be nationally representative of the U.S. population, accounting for unequal probabilities of selection and nonresponse.

^¶^ Respondents with missing or unknown values were excluded from prevalence ratio calculations.

** Calculated via use of the Rao-Scott chi-square test. The overall p-values could not be calculated (p = NC) for some characteristics with zero prevalence in categories not shown (e.g., “other” category for sexual orientation).

^††^ Relative standard error >30% but <40%.

^§§^ Relative standard error >50%; estimates are suppressed.

^¶¶^ Relative standard error >40% but <50%.

*** Ratio of family income to poverty level as defined by the U.S. Census Bureau.

^†††^ Participants who were told by a doctor or other health care professional in the last 12 months that they had chlamydia or gonorrhea or were ever told they have herpes, human papilloma virus, or genital warts.

Significant differences in the prevalence of lifetime PID were observed by the sexual behaviors and sexual health histories of respondents. The prevalence of self-reported lifetime PID among women whose age of sexual debut was <12 years was approximately eight times that of women whose age of sexual debut was ≥18 years (PR = 8.6). Similarly, the lifetime PID prevalence among women with ≥10 lifetime male vaginal sex partners was approximately three times that of women with a single partner (PR = 3.6). The prevalence of lifetime PID was approximately double in women reporting lesbian/bisexual versus heterosexual orientation (PR = 2.1), and the prevalence among women reporting a previous STI diagnosis was approximately three times that of women without a previous STI diagnosis (PR = 3.3).

In stratified analyses (Figure), the prevalence of self-reported lifetime PID among women reporting a previous STI diagnosis was similar in whites and blacks (10.0% [95% CI = 4.4–15.6] versus 10.3% [95% CI = 1.3–19.4], p = 0.97). However, among women with no previous STI diagnosis, the prevalence of self-reported lifetime PID in black women was 2.2 times the prevalence in white women (black: 6.0% [95% CI: 3.4–8.6] versus white: 2.7% [95% CI: 1.1–4.4], p = 0.01).

**FIGURE:**
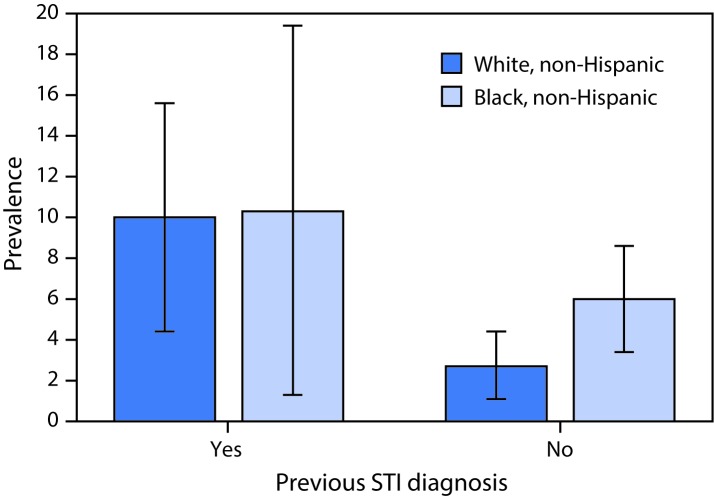
Prevalence of self-reported lifetime pelvic inflammatory disease* among sexually experienced women^†^ aged 18–44 years (n = 1,171), by race/ethnicity and previous STI diagnosis^§,¶^ — National Health and Nutrition Examination Survey, United States, 2013–2014 **Abbreviation:** STI = sexually transmitted infection. * Prevalence estimates based on response to the question, “Have you ever been treated for an infection in your fallopian tubes, uterus or ovaries, also called a pelvic infection, pelvic inflammatory disease, or PID?” Estimates were weighted to be nationally representative of the U.S. population, accounting for unequal probabilities of selection and nonresponse. ^†^ Based on a response of “Yes” to the question, “Have you ever had vaginal, anal, or oral sex?” ^§^ Participants who were told by a doctor or other health care professional in the last 12 months that they had chlamydia or gonorrhea or were ever told they have herpes, human papilloma virus, or genital warts. ^¶^ Bars indicate 95% confidence interval. Prevalence estimates among non- Hispanic black women with a previous STI diagnosis have a relative standard error >40% but <50%.

## Discussion

Based on NHANES 2013–2014 data, an estimated 2.5 million women aged 18–44 years in the United States reported a lifetime history of PID diagnosis. The increased prevalence among women reporting a previous STI diagnosis and other behaviors that increase risk for acquiring an STI underscores the need for STI prevention and control activities. The higher prevalence among black versus white women without a previous STI diagnosis suggests that black women might be more likely to have had an undiagnosed, asymptomatic STI or less likely to have received or reported a diagnosis for a symptomatic infection, possibly because of decreased access to care (*2*).

PID is not nationally notifiable but is reportable in some states. Few studies have assessed the incidence of PID using nationally representative data. Estimates from the National Survey of Family Growth found a similar prevalence of self-reported lifetime PID treatment among reproductive-aged women (5.7% during 2006–2010) and variations in self-reported lifetime PID treatment by sexual behaviors (*3*); women with a younger age of sexual debut and a higher number of lifetime vaginal sex partners were more likely to have received treatment for PID. The results of that study indicated that black race, having less than a high school education, and an income <150% of the federal poverty level were associated with receipt of PID treatment.

The findings in this study are subject to at least four limitations. First, small sample sizes led to unstable estimates and wide CIs. Hence, these results should be interpreted cautiously. Second, NHANES PID data are based on self-report, an inherent problem of which is social-desirability bias. Third, given that PID is often asymptomatic and difficult to diagnose because of the lack of a diagnostic test and the low sensitivity and specificity associated with the use of a clinical case definition, estimates in this report might underestimate the actual prevalence of PID. Finally, temporality could not be established for all factors, and as such, there is no way to know whether the occurrence of certain factors (i.e., health insurance, access to health care, previous STI diagnoses) occurred before the PID diagnosis.

PID can result from untreated bacterial infections, including chlamydia and gonorrhea, both of which are treatable and preventable. Each case of PID results in an estimated average cost of $3,202 (*4*). Chlamydia and gonorrhea are the most commonly reported STIs in the United States, with approximately 1.5 million chlamydia and approximately 400,000 gonorrhea infections reported in 2015 (*5*). Most chlamydia and gonorrhea infections are asymptomatic in women and many go undiagnosed and untreated (*6*). Results from randomized controlled trials suggest that chlamydia screening is associated with a decreased incidence of PID (*7*,*8*). The U.S. Preventive Services Task Force recommends that all sexually active women aged <25 years and older women at increased risk for infection be screened for chlamydia and gonorrhea (*9*).

Using nationally representative data, this study found a substantial prevalence of PID in the United States. Lifetime prevalence of PID was highest in women with sexual behaviors and a sexual health history putting them at increased risk for STIs, including having had a prior STI diagnosis, and differed by race/ethnicity in those without a prior STI diagnosis. Given the potential for asymptomatic infections to lead to PID and the costs associated with treatment, it is important for clinicians to adhere to U.S. Preventive Services Task Force guidelines for chlamydia and gonorrhea screening in an effort to decrease the PID burden in sexually experienced women of reproductive age nationwide (*9*).

**Summary**

**What is already known about this topic?**

Pelvic inflammatory disease (PID) has various etiologies, including untreated chlamydia and gonorrhea infections, and is a potential sequela of these infections, with serious and costly outcomes. Chlamydia and gonorrhea infections are largely asymptomatic among women, and as such, most infections are undiagnosed and untreated.

**What is added by this report?**

In the National Health and Nutrition Examination Survey 2013–2014 cycle, the prevalence of a self-reported lifetime PID diagnosis was 4.4% among sexually experienced reproductive-aged women, equating to 2.5 million prevalent PID cases in women aged 18–44 years nationwide. Prevalence of a self-reported lifetime PID diagnosis varied by sexual behaviors and sexual health history and differed by race/ethnicity in women without a prior STI diagnosis.

**What are the implications for public health practice?**

These findings highlight differences in reproductive health by sexual behaviors and sexual health history. Given the potential of asymptomatic infection to lead to PID and the substantial costs associated with treatment, it is important that clinicians follow chlamydia and gonorrhea screening recommendations for women to decrease the incidence of PID.
